# Electrospun Nanofibers of Polycaprolactone/Collagen as a Sustained-Release Drug Delivery System for Artemisinin

**DOI:** 10.3390/pharmaceutics13081228

**Published:** 2021-08-09

**Authors:** Peipei Huo, Xinxu Han, Wenyu Zhang, Jing Zhang, Parveen Kumar, Bo Liu

**Affiliations:** 1 Laboratory of Functional Molecules and Materials, School of Physics and Optoelectronic Engineering, Shandong University of Technology, Xincun West Road 266, Zibo 255000, China; h181175299@126.com (X.H.); zwy15269646616@126.com (W.Z.); jingzhang0811@126.com (J.Z.); kumar@sdut.edu.cn (P.K.); 2 School of Materials Science and Engineering, Shandong University of Technology, Zibo 255000, China

**Keywords:** artemisinin, nanofiber, anti-crystallization, drug release

## Abstract

The application of artemisinin (ART) in the treatment of malaria has been restricted to a certain degree due to its inherent limitations, such as short half-life, poor solubility, limited bioavailability, and re-crystallization. Electrospun nanofibers loaded with ART provide an excellent solution to these limitations and yield sustained drug release as well as inhibition of drug re-crystallization. In this study, ART-loaded polycaprolactone (PCL)/collagen (Col) nanofibers with different proportions of polymers were prepared. ART-loaded PCL/Col nanofibers were characterized, and further ART anti-crystallization and release behaviors were studied. SEM was used to observe the morphology of PCL/Col nanofibers. X-ray diffraction (XRD) was used to characterize the physical state of ART in ART-loaded PCL/Col nanofibers. Fourier transform infrared spectroscopy (FTIR), water contact angle measurement, weight loss, degree of swelling, and drug release experiments can verify the differences in performance of ART-loaded PCL/Col nanofibers due to different polymer ratios. The release curve was analyzed by kinetics, showing sustained release for up to 48 h, and followed the Fickian release mechanism, which was shown by the diffusion index value obtained from the Korsmeyer-Peppas equation.

## 1. Introduction

Malaria is a serious parasitic disease that has been spreading all over the world but mainly in Africa [1]. Artemisinin (ART), a peroxide-containing sesquiterpene lactone [2,3], is currently the most widely used and most effective anti-malarial drug [4,5]. It shows strong activity against malaria due to *Plasmodium falciparum* (a unicellular protozoan parasite that is the deadliest species of malaria-causing *Plasmodium* in humans) and plays a key role in the treatment and continuous control of malaria worldwide [6]. Due tribute should be given to a distinguished Chinese researcher, Nobel laureate Youyou Tu, for her original and successful separation of ART [7]. In the past two decades, ART and its derivatives (e.g., artesunate, dihydroartemisinin, artesunate) have been identified as safe, less toxic, and highly effective anti-malarial drugs [8,9,10,11]. ART not only treats malaria but also has potential anti-tumor, anti-cancer, and anti-parasite applications [12,13]. However, ART has some disadvantages, such as short half-life, poor solubility, and limited bioavailability [14]. The problem of ART lies mainly in its re-crystallinity [15], which could reduce its bioavailability [16] and therapeutic effect on the human body. Therefore, it is necessary to suppress ART crystallization to maintain its effectiveness and extend the shelf-life of its active ingredients [17].

Transdermal drug delivery systems (TDDS) are an effective way to deliver drugs through the skin of a patient. TDDS have several advantages; for example, it can control the rate and location of drug release in vivo and has high safety levels. However, the main problem with TDDS is the crystallinity of model drugs. Crystallized drugs in TDDS may lead to reduced solubility and reduced drug flux through the stratum corneum [18]. The great permeability of ART offers potential applications in gastrointestinal administration and TDDS [19]. However, its high crystallinity may prevent its use as a model drug in TDDS [20]. Studies have shown that nanofibers can inhibit ART re-crystallization and can be used as TDDS [17].

In recent years, with the rapid development of nanotechnology, nanofibers prepared by electrospinning have been widely studied as TDDS [21,22,23]. Nanofibers prepared by electrospinning can be used as drug release systems due to their high specific surface area [20]. Loading drugs or biologically active substances into a polymer by electrospinning can improve encapsulation efficiency and retain pharmacological activity [24]. At the same time, nanofibers can reduce the burst release of drugs through the proper selection of drug–polymer–solvent systems [25]. Moreover, electrospun nanofibers are favorable for TDDS due to their structural similarity with the extracellular matrix, appropriate porosity [26], and the ability to be cut into any size and shape, making them the preferable material for clinical applications in different situations [27]. Furthermore, it is reported that the rapid drying process of electrospinning is beneficial for the solid dispersion of crystalline drugs. Therefore, nanofibers prepared by electrospinning can be used to inhibit the re-crystallization of model drugs. Yu et al. prepared acyclovir-loaded core-shell nanofibers that inhibited the re-crystallization of drugs and had the potential to dissolve and disperse the poorly water-soluble drugs [28]. Shi et al. prepared ART-loaded cellulose acetate (CA)/polyvinylpyrrolido (PVP) nanofibers, in which ART re-crystallization was successfully prohibited. Additionally, CA/PVP core-shell nanofibers facilitate high amorphous ART transdermal transmission efficiency in TDDS [17].

In the electrospinning process, synthetic polymer and natural polymer are used to prepare nanofiber scaffolds. Blends of hydrophobic and hydrophilic polymers have some advantages that cannot be achieved by individual polymers [26]. Poly (ε-caprolactone) (PCL) is a synthetic polymer with high mechanical strength and excellent biocompatibility and biodegradability; it has been widely used in biomedical materials, but its highly hydrophobic nature limits its use. Collagen (Col) is a natural polymer with remarkable biodegradability, low antigenicity, high hydrophilicity, and good biocompatibility. However, its mechanical strength as a tissue engineering scaffold material is poor. Thus, the addition of hydrophilic Col could solve the problems in PCL application associated with its hydrophobic nature. Arian Ehterami et al. prepared Cs-Ins nanoparticle-loaded PCL/Col nanofibers and investigated their surface wettability, microstructure, capacity to absorb water, mechanical properties, blood compatibility, and cellular behavior. It was concluded that PCL/Col nanofibers have medical application prospects, specifically in wound dressings [26]. It was reported that the implementation of binary blend fibers (PCL and gelatin) can serve as the medium of cell proliferation [29], which further reflects that it is a promising breakthrough strategy to generate novel biomedical devices and therapeutic systems for targeted drug delivery, utilizing the biodegradable and biocompatible features. Other issues of interest include the choice of solvent and hydrolytic stability in the case of electrospinning from polymer solutions [30,31].

In the past few years, various detection methods have been developed to detect ART, such as thin layer chromatography (TLC) [32], high performance liquid chromatography (HPLC), HPLC-mass spectrometry [12]. However, most of these detection methods are cumbersome and require a lot of time. UV–visible spectrophotometry has been widely used to detect substances qualitatively or quantitatively [33]. However, the signals to detect ART using UV-visible spectrophotometers are extremely insignificant. Therefore, in order to obtain detectable spectroscopic signals for its concentration in the solution, ART is derivatized (Q292) in advance, which can present obvious electronic transition effects [33].

In this paper, the key is to develop nanofibers with potential application to TDDS that can inhibit the crystallization of ART as well. Different proportions of PCL/Col were prepared as ART carriers, and their morphology and functional characteristics were studied. Moreover, a drug release study was performed and the release kinetics analyzed. The prepared ART-loaded PCL/Col electrospun nanofibers inhibited the crystallization of ART and showed their great potential as an ART transdermal drug delivery system.

## 2. Materials and Methods

### 2.1. Materials

Artemisinin (ART, 63968-64-9, 98%), polycaprolactone (PCL, 24980-41-1, molecular weight: 80000), collagen (Col, 9001-12-1, 90%), acetic acid (64-19-7, 99.7%), sodium hydroxide (NaOH, 1310-73-2, 98%), sodium phosphate dibasic (Na_2_HPO_4_, 10039-32-4, 99%), potassium phosphate monobasic (KH_2_PO_4_, 7778-77-0, 99%), potassium chloride (KCl, 7447-40-7, 99.98%), and sodium chloride (NaCl, 7647-14-5, 99.9%) were purchased from Aladdin, China.

### 2.2. Preparation of Nanofibers

#### 2.2.1. Preparation of Precursor Solutions

PCL (10% in acetic acid (*w*/*v*)) and Col (5% in acetic acid (*w*/*v*)) solutions were blended in different mass ratios, and ART (10 wt% of total polymers) was dissolved in the mixed solutions. Drug-loaded electrospun nanofibers containing precursor solutions with different mass ratios of PCL and Col were denoted as PCL/Col-A (30%/70%, *w*/*w*), PCL/Col-B (60%/40%, *w*/*w*), and PCL/Col-C (90%/10%, *w*/*w*).

#### 2.2.2. Preparation of ART-Loaded PCL/Col Nanofibers

ART-loaded PCL/Col nanofibers were prepared by electrospinning. The electrospinning parameters used are as follows: volume of syringe, inner diameter of the needle, distance between the receiver and the needle, voltage, and pushing speed were 10 mL, 0.41 mm, 10 cm, 20 kV, and 1 mL/h, respectively; 30% humidity was maintained at room temperature throughout the electrospinning process. After the electrospinning, the ART-loaded PCL/Col nanofibers were dried in a vacuum oven at room temperature overnight.

### 2.3. Characterization

#### 2.3.1. Scanning Electron Microscopy (SEM)

In order to obtain the morphology of the ART-loaded PCL/Col nanofibers, quanta 250 field emission environment scanning electron microscopy (SEM, FEI, Hillsboro, OR, USA) was used. Each sample was sputter-coated with thin gold prior to observation using SEM.

#### 2.3.2. X-ray Diffractometer (XRD)

The pure ART, the physical mixture of ART/PCL/Col, the ART-loaded PCL/Col nanofibers, and the ART-loaded PCL/Col nanofibers after sealed storage at ambient temperature for six months were analyzed by an X-ray diffractometer (XRD, Bruker AXS, Karlsruhe, Germany), with the diffraction angle 2θ from 5° to 40°, at a scanning speed of 1° per minute at room temperature. The physical state of ART in ART-loaded PCL/Col nanofibers was examined.

#### 2.3.3. FTIR Spectroscopy

Pure ART, PCL/Col nanofibers, and ART-loaded PCL/Col nanofibers were analyzed from 4000–600 cm^−1^ using Fourier transform infrared (FTIR) spectroscopy (Nicolet5700, Waltham, MA, USA).

#### 2.3.4. Water Contact Angle Analysis

The surface hydrophilicity of ART-loaded PCL/Col nanofibers with different proportions of polymers was measured by a contact angle meter analysis system (JY-82, Chengde Dingsheng, Chengde, China).

#### 2.3.5. ART Detection

The ART was measured by a UV–vis spectrophotometer (UV-3600 plus, Shimadzu, Kyoto, Japan). The ART solution was blended with a 0.05 N NaOH solution at a ratio of 1:9 (ART: NaOH). The mixed solution was kept at 50 °C for 30 min in a water bath and then cooled to room temperature using running water to obtain a derivative of ART (Q292). Q292 can be observed with an absorption peak at 292 nm using a UV–vis spectrophotometer [30].

#### 2.3.6. Swelling and Weight Loss

The degree of swelling and weight loss of ART-loaded PCL/Col nanofibers was calculated. Both samples were measured in the phosphate buffer saline (PBS) solution at room temperature for 24 h.

Degree of swelling = (M_1_ − M_2_)/M_2_ × 100; (1)

Weight loss = (M − M_2_)/M × 100, (2)

where M is the initial mass of the electrospun nanofibers after overnight drying; M_1_ is the mass of swelled nanofibers after removing surface moisture with a filter paper; M_2_ is the mass of residual nanofibers after vacuum drying of M_1_ at room temperature until a constant weight [34].

#### 2.3.7. Drug Release

In order to study the release characteristics of ART, a standard curve for ART was drawn. A standard solution of pure ART in water/ethanol (9:1, *v*/*v*) was prepared and then derivatized. The absorbance of the solution was measured at 292 nm with a UV–vis spectrophotometer.

The release study was performed in PBS. A certain amount of ART-loaded PCL/Col nanofibers was placed in a 50 mL PBS solution at 37 °C. At regular intervals, 1 mL PBS solution was taken out while the same volume of fresh PBS was added in order to maintain the original volume. After that, the extracted solution was subjected to derivatization treatment, and absorbance was measured with a UV–vis spectrophotometer. The amounts of ART release were calculated using a standard curve and plotted against time up to 48 h. All the measurements were performed in triplicate.

#### 2.3.8. The Kinetics Analysis of Drug Release

Drug release kinetics and mechanisms are descriptive features of nano-drug carrier systems [35]. Many kinetic models have been used to study drug release properties from nanofiber carriers. In this article, the release of ART in different polymer proportions of ART-loaded PCL/Col nanofibers was measured within 48 h. The release data of ART were processed by different kinetic models to determine the release mechanism of ART in PCL/Col nanofibers. The release curve was fitted using the following kinetics.

Zero-order release kinetics:

C_t_/C_∞_ = k_0_t; (3)

First-order release kinetics:

Ln(1 − C_t_/C_∞_) = −k_1_t; (4)

Higuchi kinetics model:

C_t_/C_∞_ = kt^1/2^; (5)

Korsmeyer-Peppas model:

C_t_/C_∞_ = k_p_t^n^, (6)

where C_t_ is the drug concentration released at time t; C_∞_ is the amount of drug released at infinite time (t_∞_); k_0_, k_1_, k, and k_p_ are rate constants for zero-order, first-order, Higuchi, and Korsmeyer-Peppas kinetic models, respectively. The diffusion index *n* in the Korsmeyer-Peppas model represents the release mechanism of the drug from its carrier. If *n* = 0.5 (or is in the vicinity of this value), the release mechanism is a classical Fickian diffusion. The values in the range of 0.5 < *n* < 1 indicate a typical anomalous (non-Fickian mechanism, case II, which is the superposition of Fickian and non-Fickian processes) mechanism of release. If *n* > 1, it is the Super case II variant [35,36].

## 3. Results and Discussion

### 3.1. Morphology Characterization

**Figure 1:**
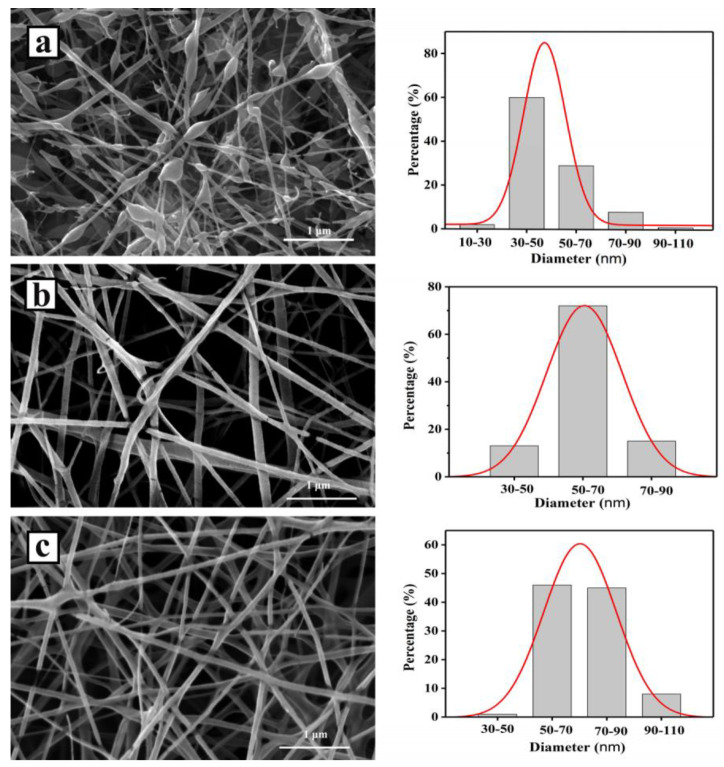
SEM images of electrospun ART-loaded PCL/Col nanofibers and nanofiber diameter distribution histograms. (**a**) PCL/Col-A; (**b**) PCL/Col-B; (**c**) PCL/Col-C.

SEM images and the diameter distribution of ART-loaded PCL/Col samples (from randomly selected 100 nanofibers) with different polymer ratios are shown in Figure 1. Before electrospinning, ART-, PCL-, and Col-blended precursor solutions were homogeneous, indicating the formation of a single-phase [37]. It can be seen in the SEM images that ART-loaded PCL/Col-A nanofibers present more beads with diameters of around 47.6 nm. As the ratio of PCL in PCL/Col nanofibers increased, the number of beads decreased. The diameters of PCL/Col-B and PCL/Col-C nanofibers were centered at 60.2 and 71.7 nm, respectively. This is because the addition of the PCL component in the precursor increases the viscosity and mass density of the precursor solution, which is beneficial to electrospinning [38,39]. Many studies have proven that in the case of electrospinning, the precursor solution jet, with low viscosity and mass density, is subjected to force stretching in the electrostatic field. If the molecular chains are not entangled or insufficiently entangled, they cannot effectively resist the action of external forces, such as stretching by the electric field force that exists in the electrospinning apparatus between the syringe and the acceptor, subsequently leading to break. In addition, due to the shrinkage of polymer molecular chains, they tend to agglomerate, and, finally, polymer beads are formed. It can be concluded that a precursor solution with appropriate mass density and viscosity is a decisive factor determining the continuous morphology of electrospun nanofibers. As the mass density and viscosity of the precursor solution increase, the degree of entanglement between the molecular chains can be sufficiently enlarged. In this case, the solution jet can sustain a longer relaxation time although it is subjected to stretching by the electric field force. The molecular chains are tangled and oriented along the axial direction of the jet, which can effectively suppress the breakage of some molecular chains in the jet, thereby forming a continuous nanofiber structure [37,40,41,42,43]. The above reasons are reflected in the SEM image: PCL/Col-A nanofibers had the most beads, and PCL/Col-C nanofibers had the fewest beads and continuous uniformity, which was due to the increase in the concentration and viscosity of the precursor solution. In summary, it can be concluded that PCL/Col C is the most suitable ratio for preparing fibers.

### 3.2. Crystallographic Structure Analysis

**Figure 2:**
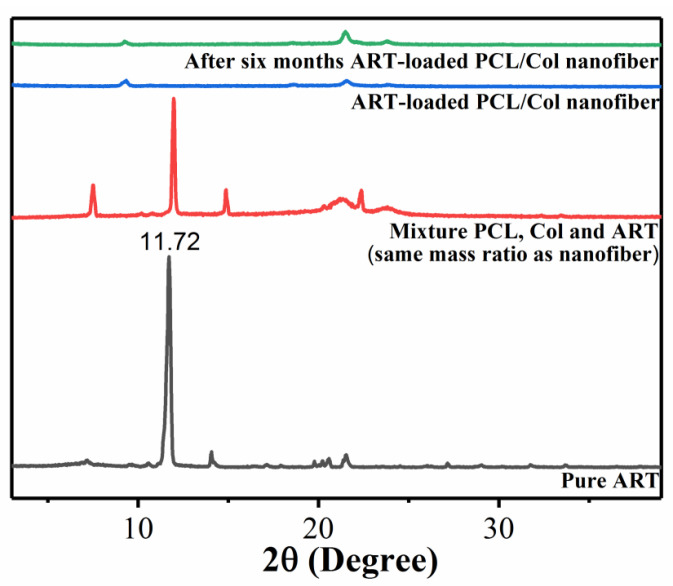
XRD patterns of neat ART, physically mixed ART/PCL/Col, ART-loaded PCL/Col nanofibers, and ART-loaded PCL/Col nanofibers after six months of storage.

**Figure 3:**
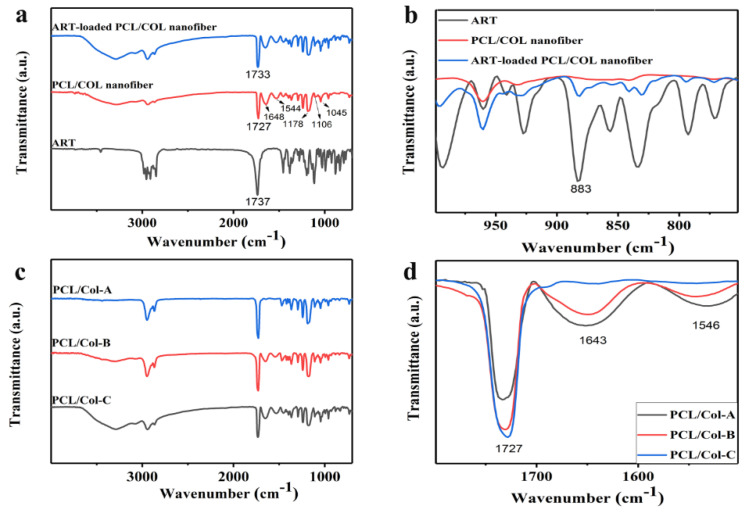
FTIR spectra of (**a**,**b**) pure ART, PCL/Col nanofibers, ART-loaded PCL/Col nanofibers; (**c**,**d**) ART-loaded nanofibers with different polymer ratios: PCL/Col-A, PCL/Col-B, and PCL/Col-C.

The physical state of ART present in nanofibers was determined by X-ray diffraction [17]. XRD patterns of pure ART, the physical mixture of ART/PCL/Col (same mass ratio as ART-loaded PCL/Col nanofibers), and ART-loaded PCL/Col nanofibers are shown in Figure 2. The XRD spectra of pure ART displayed several peaks located at 7.12°, 11.72°, 14.08°, 20.52°, and 21.5°, with the most intensive at 11.72°, indicating the highly crystalline nature of ART. However, after loading the drug into PCL/Col polymer fibers, all the characteristic peaks and the signature peak at 11.72° were completely suppressed. The result indicates that the crystallinity of ART is significantly reduced in PCL/Col nanofibers, thereby existing in an amorphous form. In order to rule out the possibility that the reduced intensity might be because of the low mass ratio of ART in the drug-loaded nanofiber system, another XRD measurement of the physical mixture of ART/PCL/Col with exactly the same mass ratio was carried out. It can be seen from Figure 3 that the characteristic peak of ART at 11.72° is still present in the physical mixture, indicating the existence of its crystalline state in the physical mixture. Thus, it proves that the disappearance of the characteristic peak of ART in ART-loaded PCL/Col nanofibers is not due to the low signal but to the amorphous state instead. The above results suggest that PCL/Col nanofibers can inhibit the re-crystallization of ART [20]. The inhibition of the re-crystallization of drugs within electrospun nanofibers via electrospinning technology is favorable for the dispersion of poorly water-soluble drugs [44]. In addition, hydrophilic polymers allow more extensive wetting of drug particles, resulting in higher solubility and dissolution rates of poorly water-soluble drugs. Moreover, combining the drug with an amorphous or less crystalline polymer can change the crystallinity of the drug. Subsequently, a less crystalline or more amorphous ART, with improved solubility and resultant enhanced bioavailability, was synthesized. To summarize, in the fast-drying electrospinning process, it is possible to “freeze” drug molecules distributed randomly in the solid polymer fiber matrix to a state comparable with that in liquid form, thus preventing the drug from re-crystallizing [45]. In order to understand the stability of ART-loaded PCL/Col nanofibers, XRD measurements were carried out after 6 months of sealed storage at ambient temperature. It is shown in the top curve of Figure 2 that the signature peak at 11.72° is still perfectly inhibited. This phenomenon illustrates that within ART-loaded PCL/Col nanofibers, ART re-crystallization was inhibited quite effectively during storage. The inhibition of ART re-crystallization can increase its bioavailability within the human body to attain a better therapeutic effect on disease.

### 3.3. FTIR Analysis

The encapsulation of drugs within nanofiber carriers can, in principle, lead to peculiar changes in FTIR spectroscopy; therefore, the comparison of FTIR spectroscopy between pure ART and ART-loaded PCL/Col nanofibers can be rather informative [46,47]. The FTIR spectroscopy of pure ART, PCL/Col nanofibers, and ART-loaded PCL/Col nanofibers are displayed in Figure 3a,b, where Figure 3b shows an enlarged view, presenting the 1000–750 cm^−1^ section of Figure 3a. It is reported that the pharmacological activity of ART is due to its peroxide bridge, which can form very active free radicals [24]. As shown in Figure 3b, the infrared spectra of both ART and ART-loaded PCL/Col nanofibers display the presence of a characteristic band at 883 cm^−1^, indicating the presence of ART in its active form in ART-loaded PCL/Col nanofibers. The characteristic peaks can be distinguished from Figure 3a: the peak at 1737 cm^−1^ corresponds to the tensile vibration of C=O in ART [48]. PCL exhibited the characteristic peaks at 1170 cm^−1^ (stretching band of C-O); 1048 cm^−1^, 1105 cm^−1^, 1220 cm^−1^ (C-O-C tensile vibration); 1727 cm^−1^ (carboxylic acid absorption). Col exhibited the characteristic peaks at 1544 cm^−1^ (amide II) and 1648 cm^−1^ (amide I) (characterized antibiotic tape) [17]. In particular, FTIR is very sensitive to hydrogen bonds, causing a redshift in the functional group due to a reduction in the frequency of vibration of the groups involved in hydrogen bond formation. In ART-loaded PCL/Col nanofibers, the peak at 1737 cm^−1^ moves toward a lower wavenumber (1733 cm^−1^), which may be due to the presence of hydrogen bond formation between the nanofibers and ART. This result is beneficial as it is reported in the literature that hydrogen bonds between the drug and polymer matrix can enhance the ability of drugs to be soluble in the polymer [49], thereby preventing drug re-crystallization in nanofibers [20].

Further, the ratio of PCL in the polymer system was studied by FTIR spectroscopy. FTIR spectra of ART-loaded PCL/Col nanofibers with different proportions of polymers are shown in Figure 3c,d. Figure 3d shows an enlarged view, presenting the 1800–1500 cm^−1^ section of Figure 3c. A peak at 1727 cm^−1^ corresponds to the carboxylic acid of PCL, and peaks at 1544 and 1648 cm^−1^ correspond to the amide group of Col. However, the intensity of these characteristic peaks changed significantly, and the specific data presenting the intensity value are shown in Table 1. The characteristic peak intensity of PCL/Col-A was assumed to be 1 and set as a reference. The ratio for the carboxylic acid peaks between PCL/Col-B and PCL/Col-A nanofibers was 1.250, whereas the ratio between PCL/Col-C and PCL/Col-A nanofibers increased to 1.315. The increased carboxylic acid peak intensity and increased peak intensity ratio suggested a higher PCL percentage in the order of PCL/Col-A < PCL/Col-B < PCL/Col-C. With a decrease in the proportion of Col in PCL/Col nanofibers, the amide characteristic peak of Col decreases. The ratio of the amide I peak (1648 cm^−1^) between PCL/Col-B and PCL/Col-A nanofibers decreased to 0.754, and the ratio between PCL/Col-C and PCL/Col-A nanofibers further decreased to 0.079. In contrast, the ratio of the amide II (1544 cm^−1^) peak between PCL/Col-B and PCL/Col-A nanofibers decreased to 0.638, and the ratio between PCL/Col-C and PCL/Col-A nanofibers further decreased to 0.133. The decrease in the amide I and amide II group signals in the infrared spectrum correspond to a decrease in Col content in nanofibers. The result is consistent with literature reports [48]. All these results show that ART was successfully loaded into PCL/Col nanofibers, leading to physical interactions between ART and the nanofiber matrices. At the same time, the ratio of the characteristic peak changed as the proportion of polymer in the nanofiber matrices was varied.

### 3.4. Water Contact Angle Analysis

**Figure 4:**
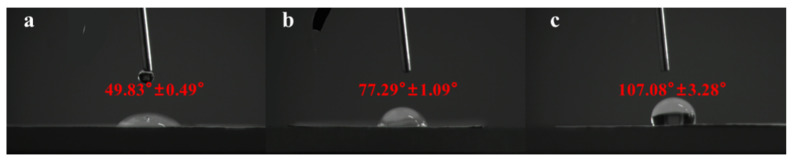
The contact angle (CA) measurement of ART-loaded PCL/Col nanofibers with different polymer ratios: (**a**) PCL/Col-A; (**b**) PCL/Col-B; (**c**) PCL/Col-C.

The hydrophilicity of the surface of biomedical materials is a key factor determining cell adhesion and cell diffusion [50]. Therefore, the contact angle of the drug-loaded nanofibers should be measured to determine their hydrophilicity for verifying their release properties. If the contact angle is greater than 90°, the material is hydrophobic, and if the contact angle is less than 90°, the material is hydrophilic [51]. The contact angles of ART-loaded PCL/Col nanofibers with different ratios are shown in Figure 4. The contact angles of PCL/Col-A and PCL/Col-B were found to be 49.83 ± 0.49° and 77.29 ± 1.09°, respectively, indicating hydrophilicity in both the materials. However, the contact angle of 107.08 ± 3.28° for PCL/Col-C suggests its hydrophobic nature. An increase in the proportion of PCL in PCL/Col nanofibers caused an increase in hydrophobicity, and the corresponding change was also seen from the infrared spectrum. The different ratios of PCL in the ART-loaded PCL/Col nanofibers led to varied hydrophilicity of the materials. Therefore, the ratio of PCL in the nanofiber matrix can be varied to further adjust the hydrophilicity/hydrophobicity of nanofibers. To this point, it is reasonable to expect that a good sustained-release effect of ART can be achieved by adjusting the ratio of PCL in the nanofiber matrix.

### 3.5. Degree of Swelling and Weight Loss

**Figure 5:**
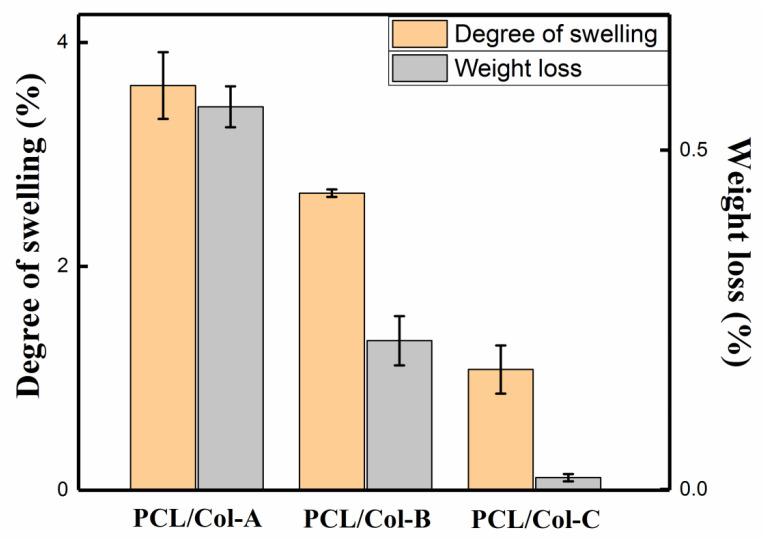
The degree of swelling and weight loss of the ART-loaded PCL/Col nanofibers with different polymer ratios.

Figure 5 shows the degree of swelling and weight loss of ART-loaded PCL/Col nanofibers prepared in different polymer ratios at room temperature for 24 h. Degree of swelling and weight loss are two important factors that contribute to drug release [34]. As the proportion of PCL in PCL/Col nanofibers increased, the degree of swelling decreased from 3.61% (PCL/Col-A) to 1.08% (PCL/Col-B). This phenomenon occurred because, with the increase of the PCL content in the PCL/Col nanofibers, the hydrophilicity of the nanofibers decreased, resulting in a slower degradation rate of the PCL/Col nanofibers. With the increase in the PCL ratio in PCL/Col nanofibers, the weight loss decreased from 0.56% (PCL/Col-A) to 0.017% (PCL/Col-B). The decrease in weight loss can be attributed to the increase in the PCL ratio in PCL/Col nanofibers, which leads to a decrease in the number of nanofiber beads and a trend towards uniformity, which can also be seen from the SEM image. At the same time, the increased ratio of PCL in PCL/Col nanofibers restricts the entry of water into the nanofibers, causing difficulty in the solubilization of ART and Col in water. Overall, the above phenomenon was caused by the increase in hydrophobic PCL content in the PCL/Col nanofibers [52]. As the proportion of PCL in PCL/Col nanofibers increased, the hydrophilicity of the nanofibers decreased and the hydrophobicity increased, which may slow down the release of ART from ART-loaded PCL/Col nanofibers.

### 3.6. Kinetics Analysis of Drug Release

**Figure 6:**
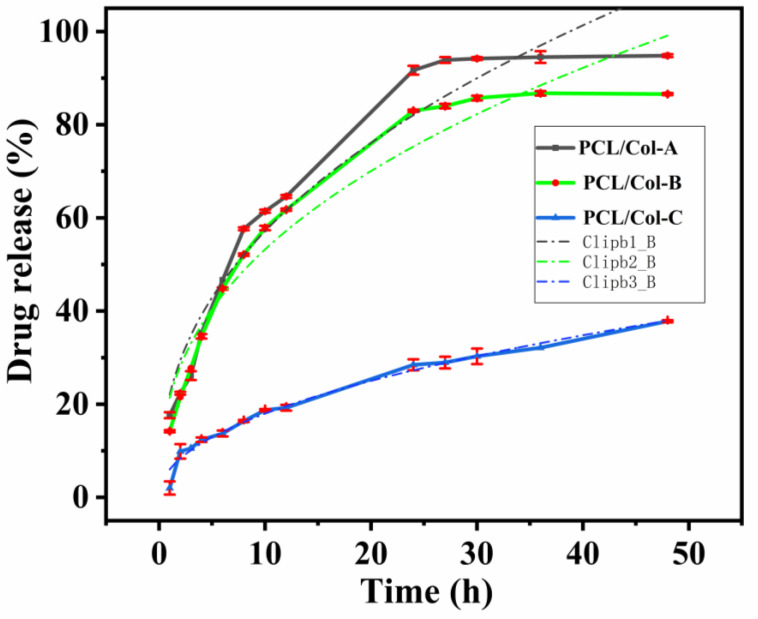
The drug release profile of the ART-loaded PCL/Col nanofibers with different polymer ratios.

As PCL is a highly hydrophobic polymer and Col is a highly hydrophilic polymer, the ratio of PCL and Col in the fiber matrix can be adjusted (as shown in the water contact angle experiment) to control the hydrophilicity of PCL/Col nanofibers in order to achieve a sustained release of ART. The release behavior of ART-loaded PCL/Col nanofibers in PBS solution was studied within 48 h. The release profiles of ART-loaded PCL/Col nanofibers with different ratios are shown in Figure 6. The ART release curves of PCL/Col-A and PCL/Col-B nanofibers presented similar release stages and were divided into three phases, namely, the burst release phase, the non-linear monotonic release phase, and the final slow release phase. The burst release phase was due to the quick release of amorphous ART present on the surface of the PCL/Col nanofibers. Subsequently, the ART present inside the PCL/Col nanofibers gradually diffused onto the nanofiber surface and then diffused into the medium solution, resulting in a slow diffusion rate [17]. In comparison, the curve PCL/Col-C, with the highest content of PCL, presents a much more relevant profile, including only two stages, whilst the second one occurs with an actual constant rate of delivery, displaying the characteristics of zero-order kinetics. However, the release rates of the three profiles showed pronounced differences. ART-loaded PCL/Col-A nanofibers exhibited the fastest release rate. The initial burst release of ART is observed in Figure 6; it quickly released up to 57.7% of the ART within 8 h, with an average rate of 7.2% h^−1^. After that, the ART release rate slowed down and released up to 93.9% within 27 h, during which the release behaves as a continuous release stage. Finally, the release is almost complete, and the profile displays as a saturated platform. The total release percentage was measured to be 94.8%, with an average release rate of 2.0% h^−1^ until the finishing point (48 h) of this experiment. On the other hand, the release of ART from ART-loaded PCL/Col-B nanofibers was relatively slower compared to PCL/Col-A nanofibers. First, the burst release phase of ART-loaded PCL/Col-B nanofibers quickly released 44.8% of the ART within 6 h, followed by a reduced release rate in which 85.7% of the ART was released in 30 h; later, a sustained release phase was observed, where 86.6% of total ART was released within 48 h, with an average release rate of 1.8%/h. However, ART-loaded PCL/Col-C nanofibers exhibited the slowest release rate. The release rate of ART from PCL/Col-C nanofibers was remarkably reduced to 9.9% in 2 h, even during the explosion phase. Finally, the release progressed in a long and slow manner, with a 37.8% reduction after 48 h and an average release rate of 0.8% h^−1^.

From the above experimental results, it can be seen that the ratio of PCL and Col in ART-loaded PCL/Col nanofibers can regulate the release performance of ART. With an increase of the hydrophobic PCL component, a slow release of ART in PCL/Col nanofibers was observed. The FTIR corresponding peak intensity changed regarding the characteristic peaks of PCL at 1727 cm^−1^ and Col at 1544 and 1648 cm^−1^; the enlargement of the water contact angle and the decreased weight loss and swelling degree test all indicated a similar conclusion.

Several kinetic models have been used to explore the mechanism of drug release from nanofiber carriers [53]. Zero-order, first-order, Higuchi, and Korsmeyer-Peppas kinetic models have been used to study the ART release mechanism from ART-loaded PCL/Col nanofibers. The relevant coefficients, such as k, R^2^, and *n*, are listed in Table 2.

According to the fitting data of several models, the Korsmeyer-Peppas model has the highest correlation (higher R^2^ value) with the actual release performance of different proportions of ART-loaded PCL/Col nanofibers. For multi-stage curves (PCL/Col-A and PCL/Col-B), the release data of all the stages (from 0 h to 48 h) were considered when mathematically processed with kinetic models. The higher the R^2^ value of the kinetic model, the more the release curve conforms to that kinetic model. R^2^ values for the Korsmeyer-Peppas model for PCL/Col-A, PCL/Col-B, and PCL/Col-C nanofibers were found to be 0.94, 0.94, and 0.98, respectively, which are closer to 1 and higher than other kinetic models. The results show that regardless of the polymer ratio, the release of ART from all the PCL/Col nanofibers followed the Korsmeyer-Peppas model. Moreover, the diffusion exponent value (*n*) from the fitting data of the Korsmeyer-Peppas model applied in the release curve of ART released from PCL/Col-A, PCL/Col-B, and PCL/Col-C was 0.41, 0.40, and 0.48, respectively. The value of *n* determines the drug release mechanism in drug-loaded nanofibers, and the results suggest that release occurred through Fickian diffusion, indicating that ART release is controlled by diffusion throughout the release process.

Based on the above analysis, it is speculated that the release process of ART from ART-loaded PCL/Col nanofibers exhibits the following mechanism. First, ART molecules diffuse into the PBS medium from the surface of the PCL/Col nanofibers and form holes on the surface of the nanofibers. As the release progresses, pores are gradually formed inside the nanofibers, and the medium gradually penetrates the nanofibers. Subsequently, the ART embedded in the PCL/Col nanofibers gradually dissolves and eventually completely dissolves in the medium, leaving the nanofiber scaffolds [54,55].

## 4. Conclusions

ART-loaded PCL/Col nanofibers with different polymer ratios were prepared by electrospinning technology. The morphology of the ART-loaded PCL/Col nanofibers was observed by SEM, and the nanofiber diameter was determined. The results showed that as the proportion of PCL in the PCL/Col nanofibers increased, the nanofiber diameter increased and the beading decreased. XRD analysis showed that ART existed in an amorphous form in the ART-loaded PCL/Col nanofibers, indicating that the nanofibers could inhibit the crystallization of ART. FTIR spectroscopy showed that ART was successfully loaded in the PCL/Col nanofibers. FTIR characterization of ART-loaded PCL/Col nanofibers with different polymer ratios showed that the signal of the characteristic bands in the ART-loaded nanofibers varied with the polymer content. Signal values of carboxylic acid and amide groups varied with the ratio of PCL and Col in ART-loaded PCL/Col nanofibers. The water contact angle test showed changes in the hydrophilicity and hydrophobicity of the PCL/Col nanofibers. With an increase in the content of PCL in the PCL/Col nanofiber matrix, the hydrophilicity of the nanofibers decreased, whereas the hydrophobicity increased. PCL/Col nanofibers with a PCL and Col ratio of 9:1 were hydrophobic. The degree of swelling and weight loss experiments proved the effect of the change in the content of PCL on the properties of PCL/Col nanofibers. With an increase in PCL content in the PCL/Col nanofiber matrix, the degree of swelling and weight loss decreased. The drug release experiment further verified the hydrophilic and hydrophobic variations of the PCL/Col nanofibers, and a sustained release behavior was observed. With an increase in the proportion of PCL in the matrix of PCL/Col nanofibers, the slow-release effect of ART is more promising. The kinetic analysis results showed that the release of ART from PCL/Col nanofibers was more consistent with the Korsmeyer-Peppas model. The release of ART from ART-loaded PCL/Col nanofibers occurred through Fickian release. Finally, ART re-crystallization can be significantly inhibited within the electrospun PCL/Col nanofiber system, and sustained release performance can be realized by adjusting the proportions of hydrophilic/hydrophobic polymers. It is expected that the therapeutic effect of ART can be enhanced by means of less crystalline and a sustained release manner in vivo. In this way, electrospun nanofibers have shown promising potential as future candidates in drug delivery systems.

## Figures and Tables

**Table 1:** The change in peak intensity between ART-loaded PCL/Col nanofibers with different ratios.

	Carboxylic Acid (1727 cm^−1^)	Amide I (1648 cm^−1^)	Amide II (1544 cm^−1^)
PCL/Col A	1	1	1
PCL/Col B	1.250	0.754	0.638
PCL/Col C	1.315	0.079	0.133

**Table 2:** The release models of ART-loaded PCL/Col nanofibers with different polymer ratios.

ART-Loaded PVA/Col Nanofibers	PCL/Col A		PCL/Col B		PCL/Col C	
Korsmeyer-Peppas model	k_p_	22.07 ± 2.62	k_p_	21.37 ± 2.30	k_p_	6.00 ± 0.45
	*n*	0.41 ± 0.04	*n*	0.40 ± 0.03	*n*	0.48 ± 0.02
	R^2^	0.94	R^2^	0.94	R^2^	0.98
Zero-order model	k_0_	1.80 ± 0.26	k_0_	1.59 ± 0.24	k_0_	0.67 ± 0.06
	R^2^	0.81	R^2^	0.8	R^2^	0.91
First-order model	k_1_	0.07 ± 0.007	k_1_	0.05 ± 0.005	k_1_	0.009 ± 0.0006
	R^2^	0.91	R^2^	0.9	R^2^	0.95
Higuchi model	k	16.77 ± 0.60	k	15.40 ± 0.56	k	5.57 ± 0.10
	R^2^	0.92	R^2^	0.91	R^2^	0.98
